# Identification of Novel Genes and Pathways Regulating SREBP Transcriptional Activity

**DOI:** 10.1371/journal.pone.0005197

**Published:** 2009-04-21

**Authors:** Sandipan Chatterjee, Joseph D. Szustakowski, Nirmala R. Nanguneri, Craig Mickanin, Mark A. Labow, Axel Nohturfft, Kumlesh K. Dev, Rajeev Sivasankaran

**Affiliations:** 1 Developmental and Molecular Pathways, Novartis Institutes for BioMedical Research, Cambridge, Massachusetts, United States of America; 2 Division of Basic Medical Sciences, St. George's University of London, London, United Kingdom; 3 Department of Anatomy, University College Cork, Cork, Ireland; University of Hong Kong, China

## Abstract

**Background:**

Lipid metabolism in mammals is orchestrated by a family of transcription factors called sterol regulatory element-binding proteins (SREBPs) that control the expression of genes required for the uptake and synthesis of cholesterol, fatty acids, and triglycerides. SREBPs are thus essential for insulin-induced lipogenesis and for cellular membrane homeostasis and biogenesis. Although multiple players have been identified that control the expression and activation of SREBPs, gaps remain in our understanding of how SREBPs are coordinated with other physiological pathways.

**Methodology:**

To identify novel regulators of SREBPs, we performed a genome-wide cDNA over-expression screen to identify proteins that might modulate the transcription of a luciferase gene driven from an SREBP–specific promoter. The results were verified through secondary biological assays and expression data were analyzed by a novel application of the Gene Set Enrichment Analysis (GSEA) method.

**Conclusions/Significance:**

We screened 10,000 different cDNAs and identified a number of genes and pathways that have previously not been implicated in SREBP control and cellular cholesterol homeostasis. These findings further our understanding of lipid biology and should lead to new insights into lipid associated disorders.

## Introduction

Disruption of intracellular cholesterol metabolism and trafficking is the primary cause of numerous human disorders [1]. It has been shown that the sterol regulatory element binding protein (SREBP) pathway is the master regulator of intracellular lipid homeostasis [2], [3]. SREBPs are generated from two genes, SREBF1 and SREBF2, that are transcribed to form a number of different mRNA and protein species [4]–[8]. The prevalent isoforms are SREBP-1a, SREBP-1c and SREBP-2 [9], [10], but additional splice versions have been described [4], [5], [7], [11], [12]. SREBP-1a and SREBP-1c are both transcribed from the SREBF1 gene and differ in their first and last two exons, while SREBP-2 is the predominant protein produced from the SREBF2 gene [8], [13].

SREBPs are synthesized as inactive precursors that are anchored in the membrane of the ER through two transmembrane domains [14]. The N-terminal domain contain motifs required for dimerization, DNA binding and transactivation [15], [16]. The C-terminal domain of SREBP precursors mediates the formation of complexes with SREBP cleavage-activating protein (SCAP) [17], a membrane protein important for SREBP stability and regulation [18]–[22]. Interaction of SCAP with the COPII machinery leads to the incorporation of the SCAP/SREBP complex into vesicles and transport to the Golgi [20], [23]–[25]. SREBPs are then cleaved by Site-1 and Site-2 proteases (S1P and S2P), leading to the transfer of active transcription factors to the nucleus [26]–[29]. Here, SREBP dimers bind to sterol regulatory elements (SRE) which are present in the promoter regions of genes such as low-density lipoprotein receptor (LDL-R), 3-hydroxy-3-methylglutaryl Coenzyme A reductase (HMGCR), and fatty acid synthase, and multiple other genes involved in the regulation of intracellular lipid metabolism [30], [31]. Thus, regulation of SREBP cleavage and activity is vital for cellular lipid homeostasis and cell survival.

Studies with CHO cells and mice expressing dominant positive versions of SREBPs have shown that the target genes of SREBP-1a and SREBP-2 are largely overlapping. However, SREBP-1a is somewhat more potent at activating genes involved in fatty acid synthesis while SREBP-2 has a preference for genes involved in the biosynthesis of cholesterol. The LDL receptor is controlled equally by both transcription factors [30], [31], [32]. SREBP-1c also controls fatty acid-raising genes and, although significantly weaker than SREBP-1a [30], [32], it is the predominant SREBP isoform in many tissues and in liver regulates the conversion of carbohydrates to triacylglycerol in response to insulin [33].

SREBP-1a and SREBP-2 are subject to negative feedback regulation by cholesterol [34]. Upon binding to cholesterol SCAP undergoes a conformational change that triggers its interaction with one of two ER membrane proteins termed insulin-induced gene(INSIG)-1 and INSIG2 [21], [35]–[40]. Under these circumstances SCAP dissociates from COPII, the SCAP/SREBP complex remains in the ER, and proteolytic activation is blocked [41], [42]. In another feedback loop SREBP-1a and SREBP-1c are suppressed by polyunsaturated fatty acids (PUFA) [43]–[45]. SREBP-1c transcription in the liver is controlled by liver X receptors (LXR), whose activation in turn is blocked by PUFA [43], [46].

In spite of the current research efforts in this field, our knowledge of intracellular cholesterol trafficking and homeostasis is far from complete. To gain a better handle on these events, we performed a genome-wide cDNA over-expression screen to identify modulators of SREBP activity. We used a cell-based luciferase assay that measures expression from an SREBP-specific promoter. We also performed secondary biological assays to further validate these hits. Additionally, employing a novel modification of Gene Set Enrichment Analysis (GSEA) we performed a pathway analysis on the high throughput screening data, as GSEA was originally developed for analyzing microarray experiments [47]. GSEA applies *a priori* biological knowledge to genome-scale data sets to implicate pathways in the biological system of interest [47]. In addition to known pathways regulating lipid metabolism, such as the SREBP and nuclear hormone receptor pathway, our analysis has led to the identification of a number of pathways previously not associated with the regulation of cellular cholesterol homeostasis. The data suggests that pathways involved in intracellular signal transduction such as tyrosine kinase signaling, G-protein / small GTPase pathways and ephrin signaling positively affect intracellular cholesterol homeostasis, while pathways acting at the extracellular level, such as matrix proteins, cell-matrix and cell-adhesion proteins, and pathways involved in cell structure and organization, negatively regulate cellular cholesterol homeostasis.

We have validated the results of the primary screen through a series of secondary biological assays and find considerable overlap between the genes identified by secondary screening and the pathways identified via GSEA, indicating that pathway-centric analyses of biological screening data is a valid approach that may assist in target identification. Our results implicate multiple novel genes and pathways in intracellular cholesterol homeostasis and open up novel venues for the interrogation of lipid biology and lipid-linked disease.

## Results

### Optimization of the SREBP signaling assay

**Figure 1:**
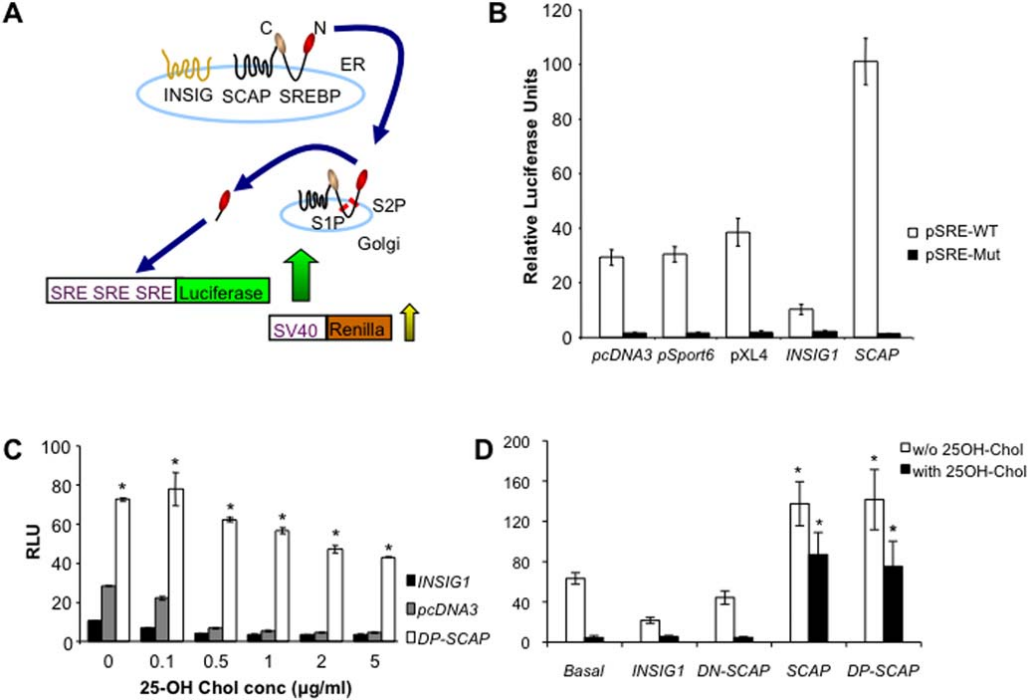
The SREBP cleavage assay. (A) Schematic representation of the SREBP cleavage assay. (B) Activity of wild-type (WT) versus mutant (Mut) SRE promoter. HEK-293 cells were set up in a 96-well plate (in triplicate). After 24 hours, cells were transfected with either WT (open bars) or mutant (black bars) luciferase reporter constructs, along with renilla luciferase construct and the indicated plasmid /cDNA. Cells were grown for an additional 24 hours before performing the assay. (C) Effects of 25-hydroxy cholesterol (25-OH chol.) on SREBP signaling. The assay was carried out under varying 25-OH cholesterol concentrations (0.1–5 µg/ml) and for different incubation periods (6, 12, and 24 hours). 25-OH chol. was added to cells 1 day after transfecting with the reporter plasmids, SRE-luciferase and renilla luciferase. Maximum suppression of SRE-luciferase signals was observed after 24 hours of incubation with 25-OH chol (shown here). The effects of DP-SCAP under high 25-OH cholesterol levels are significantly higher at all concentrations (Student's t-Test, p<0.005). (D) Effects of known repressors, activators and high cholesterol (25OH chol., 1 µg/ml) on the SREBP signaling pathway. The assay was carried out as in B. Basal refers to pcDNA3 overexpression. SCAP and DP-SCAP significantly activate the assay in the absence (white bars) or presence (black bars) of 25-OH cholesterol (Student's t-Test, p<0.05). (B–D) Error bars indicate standard deviations (n = 3). Where not visible, error bars are smaller than symbols. The graphs are representative of at least 2 independent experiments.

The reporter gene assay used in this study has been previously described [18]. Briefly, this assay is based on endogenous SREBP-mediated activation of a promoter containing three sterol regulatory elements (SREs) driving the expression of a firefly-luciferase gene (reporter construct, Figure 1A). As a transfection control for the luciferase assays, a renilla-luciferase gene, driven by a weak constitutive active SV-40 promoter, was co-transfected along with the firefly-luciferase gene (Figure 1A). The activity of the reporter gene assay was measured as a ratio between the firefly and renilla luciferase levels. Thus, a high luciferase ratio indicates SREBP pathway activation (due to a higher firefly luciferase levels) and vice-versa. For our experiments, this SREBP signaling assay was optimized by a series of steps. First, in order to use an optimal reporter construct the 3×SREs cassette was sub-cloned and tested in a number of luciferase vectors including, pGL3-Basic and pTransLucent. In our hands, the pTransLucent vector displayed higher luciferase ratios and higher signal-to-noise ratio [48] in a 384-well format and was chosen for further experiments (data not shown). Second, two mammalian cell lines HEK-293 and HeLa were tested for cell line of choice. HEK-293 cells displayed higher assay reproducibility, luciferase signals and fold change under different experimental conditions and were thus chosen for this study (data not shown). Third, a mutant SRE promoter [49] driving a luciferase gene was generated and used as a specificity control for our experiments. This mutant SRE-luciferase construct was inactive under all experimental conditions (Figure 1B). Fourth, to optimize the repression of SREBP signaling by cholesterol, a concentration response curve for 25-hydroxy (25-OH) cholesterol with varying times of incubation was performed. The result showed that incubating cells with 1 µg/ml 25-OH cholesterol for 24 hours was sufficient to repress the assay as efficiently as using 5 µg/ml 25-OH cholesterol and hence the lower concentration was used for further studies (Figure 1C).

We tested the robustness and sensitivity of the assay by evaluating the effects of *SCAP* and *INSIG1* overexpression on SREBP signaling under normal cell culture conditions (cells grown in medium containing 10% serum and antibiotics). Full-length plasmids encoding hamster *SCAP* [18] and human *INSIG1* [40] were co-transfected along with the wild-type SRE-luciferase reporter and changes in luciferase ratios were measured. We noted an approximate three and a half fold activation or repression of basal (empty vector over-expression) SREBP activity in the presence of SCAP or INSIG1, respectively (Figure 1B). A dominant-positive form of SCAP (DP-SCAP) which no longer binds INSIG1 as it contains a point mutation in its INSIG1 interacting domain [24], was equally active in enhancing SREBP signaling as wild-type SCAP. In addition, a dominant negative form of SCAP (DN-SCAP) which lacks the INSIG-binding domain [50], repressed SREBP cleavage as efficiently as over-expression of INSIG1 (Figure 1D). Next, we examined the effects of SCAP and INSIG1 over-expression in the presence of high cholesterol (1 µg/ml 25-OH cholesterol). The repressed luciferase levels found under high cholesterol conditions were rescued by the over-expression of positive components of the SREBP pathway such as wild-type SCAP or DP-SCAP as expected (Figure 1D). Under these conditions of repressed luciferase activity, we found no further measurable inhibitory effects of INSIG1 (Figure 1D).

### Genome-wide screen for regulators of cellular cholesterol homeostasis

**Figure 2:**
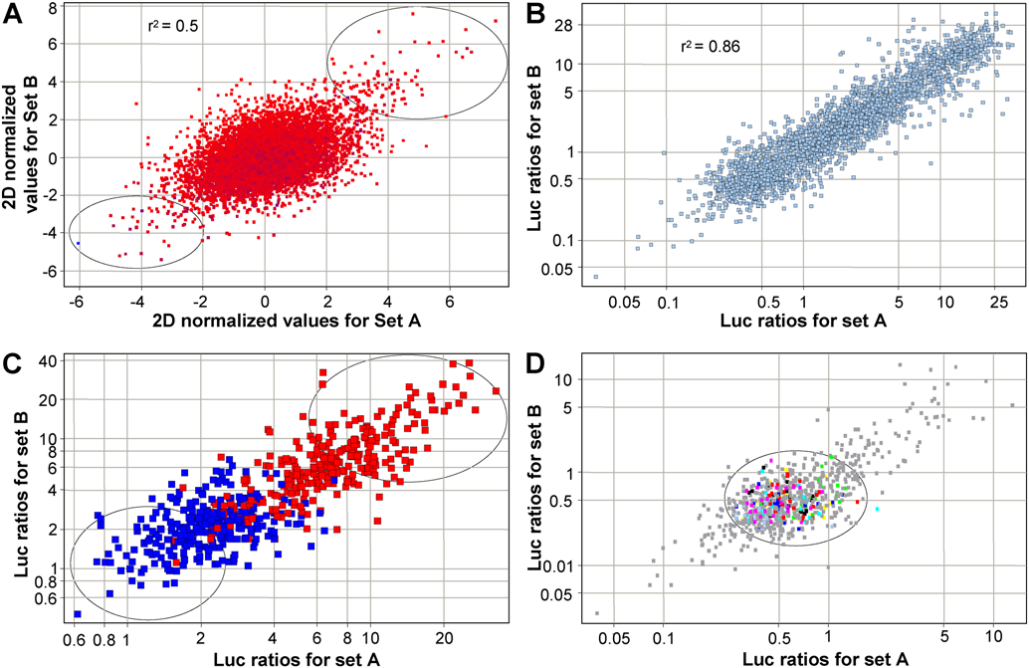
Primary and secondary screen results. (A) Scatter plot showing the result from the primary screening of 10,000 putative full-length human cDNA's in the SREBP cleavage assay. The 2D-normalized z-scores for a clone in the first experiment (x-axis) are plotted against that obtained in the second experiment (y-axis). Genes at the top right corner represent potential activators of SREBP signaling, while those at the bottom left corner represent potential repressors of SREBP signaling (circles). (B) Scatter plot representing the combined data from all secondary screens. Each clone was re-tested in triplicate in two separate experiments. Firefly to renilla luciferase ratios for a clone in the first experiment (x-axis) were plotted against the ratios for the same clone in the second experiment (y-axis). (C) Analysis of the selected 176 clones under conditions identical to those used in the primary screen. Scatter plot shows luciferase ratios obtained for a clone in the first experiment against ratios obtained for the same clone in the second experiment. Activators are represented in red and repressors in blue. Circles represent clones displaying the highest activation or repression of luciferase ratios. (D) Effect of the 176 selected activators and suppressors (grey points) on mutant SRE promoter. The scatter plot shows the luciferase ratios obtained for a clone in the first experiment against luciferase ratios obtained in the second experiment. Central data points (circle) represent genes that did not have an effect on the mutant SRE-luciferase. Grey points falling at the extremities represent clones that activated the mutant SRE-luciferase or had higher levels of renilla luciferase. Control genes are color coded as: red, DN-SCAP; dark blue, DP-SCAP; yellow, INSIG1; black, SCAP; green, pSport6; sky blue, pXL4; pink, pcDNA3.

Having determined the optimal conditions for the SREBP signaling assay, we made use of the sufficient fold difference under normal cell culture conditions to identify novel activators and repressors of the SREBP pathway. To this end, a collection of 10,000 random full-length human cDNAs was screened using a ‘gene-by-gene’ unbiased assay. The screen was carried out in duplicate so that the data could be subjected to 2-dimensional (2D) normalization i.e. normalization to remove both well-to-well and plate-to-plate variation (see Materials and Methods for details). A scatter plot for the primary screen was obtained by plotting the 2D normalized luciferase ratios for a clone in the first experiment against that obtained in the second experiment (Figure 2A). The clones lying at the extremities displayed highest activity and were selected for further validation (circles). Clones which modulated luciferase ratios by at least 2-fold were re-tested for their effects in the SREBP signaling assay. With this cut-off, a total of 176 activators and repressors were selected for re-confirmation assays. Each clone was assayed in triplicate for all the subsequent follow-up experiments. The scatter plot of the total re-screening data showed that the clones lie along the diagonal, indicating internal consistency of the experimental conditions (Figure 2B). The first of these experiments confirmed the behavior of each clone under identical conditions to that used for the original gene-by-gene unbiased screen. Furthermore, we found that genes identified in the primary screen as activators (red) clustered separately from the repressors (blue), confirming the reproducibility of the results (Figure 2C). Clones showing no clear discrimination as either activators or repressors were removed from final analysis (Figure 2C, central overlapping red and blue points and Figure S1). Genes that activated or suppressed SREBP cleavage to the greatest extent were found at the extremities of the scatter plot.

We next utilized the mutant SRE-luciferase reporter to identify non-specific regulators of SRE-luciferase. When compared to the internal controls (colored central points), a set of genes that significantly altered renilla levels (data not shown) and/or changed mutant-SRE promoter activity (Figure 2D, extremities of the scatter plot) were discarded as being false positives. Genes in the activator set that did not affect the mutant SRE promoter were deemed as candidates that regulate SREBP signaling (Figure 2D, circled central grey points). Thus, starting from 176 clones this analysis resulted in 27 activators and 40 repressors (Tables S1, S2) that showed specific effects in regulating the SREBP assay (Figure S1), while not affecting the mutant SRE promoter.

### Gene set enrichment analysis (GSEA) of high throughput screening data

**Figure 3:**
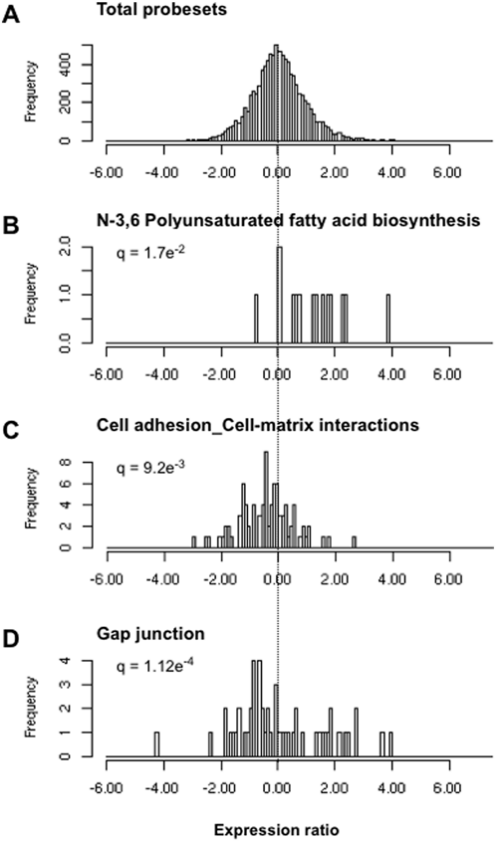
Gene set enrichment results. Distribution of 2D normalized z-scores (NZ2D) for (A) all cDNA clones used in this screen and clones assigned to the (B) N-3,6 Polyunsaturated fatty acid biosynthesis, (C) Cell Adhesion / Cell Matrix Interaction and (D) Gap Junction pathways. The rightward shift of NZ2D scores among fatty acid synthesis genes (B) relative to background (A) indicates that overexpression of genes in this pathway on balance tend to activate SREBP transcriptional activity, whereas the leftward shift in (C) indicates that the cell adhesion/cell matrix tend to inhibit SREBP transcriptional activity in this screen. Gap junction genes were spread on either side of the median (D) indicating that a sub-set of gap junction genes activated the SREBP pathway, while others repressed it.

Results from the primary gene-by gene screen were analyzed by a novel application of the Gene Set Enrichment Analysis (GSEA) technique modified for high throughput screening data (for details see Materials and Methods). We identified a number of pathways whose members coordinately modulate SREBP activity as measured in this screen. The GSEA results included pathways which are known to positively regulate intracellular cholesterol homeostasis, such as polyunsaturated and unsaturated fatty acid biosynthesis (Figure 3B) and sphingolipid metabolism pathways, as well as the nuclear hormone receptor pathway (Table 1). Additionally, signaling pathways relating to heterotrimeric G-proteins, small GTPases (including the Rab family of GTPases), RAS- and RAS-related GTPases and angiotensin signaling via PYK2, all of which have been implicated in the regulation of intracellular cholesterol metabolism (see discussion), were identified as activators of SREBP signaling. We also identified pathways previously not associated with the regulation of lipid homeostasis including ephrin signaling and epidermal growth factor receptor (EGFR) signaling pathways. In contrast to the identified activators, a majority of which impacted intracellular signaling events, the repressors from our screen were enriched for pathways associated with the extracellular matrix, cell adhesion & cell matrix interactions (Figure 3C) and matrix glycoproteins (Table 2). Proteins regulating the cytoskeleton and cell architecture and serine proteases were also found to repress the cholesterol pathway.

**Table 1:** List of pathways designated as activators of SREBP signaling by Wilcoxon test.

Pathway name	Pathway source	Probestes	Wilcoxon p-value	Wilcoxon FDR q-value
Hyperplasia	MetaCore	14	2.80E-05	0.012
Tyrosine protein kinase	PANTHER	52	3.04E-05	0.012
n-3,6 Polyunsaturated fatty acid biosynthesis	MetaCore	14	1.11E-04	0.018
Angiotensin signaling via PYK2	MetaCore	38	1.74E-04	0.023
G-protein	PANTHER	118	2.16E-04	0.023
Epidermal cell differentiation	MetaCore	64	2.21E-04	0.023
Ephrins signaling	MetaCore	46	2.44E-04	0.023
Sphingolipid metabolism	MetaCore	17	2.50E-04	0.023
Unsaturated fatty acid biosynthesis	MetaCore	11	3.30E-04	0.027
T-cell activation	PANTHER	53	3.43E-04	0.027
Small GTPase	PANTHER	97	3.96E-04	0.028
Pancreatic neoplasms	MetaCore	111	4.28E-04	0.029
Lupus erythematosus, systemic	MetaCore	38	4.37E-04	0.029
Pituitary diseases	MetaCore	17	4.45E-04	0.029
Immunoglobulin receptor family member	PANTHER	37	5.07E-04	0.032
RAS-related GTPase	PANTHER	55	5.47E-04	0.033
Neoplasms, complex and mixed	MetaCore	38	5.49E-04	0.033
Nuclear hormone receptor	PANTHER	20	6.27E-04	0.035
Axon guidance	KEGG	87	6.75E-04	0.036
EGFR signaling via small GTPases	MetaCore	24	7.03E-04	0.036
Ras-GDP/GTP	PANTHER	6	9.56E-04	0.040
Non-receptor tyrosine protein kinase	PANTHER	21	9.84E-04	0.040
EGF signaling pathway	MetaCore	39	1.03E-03	0.040
RAC1 in cellular process	MetaCore	18	1.16E-03	0.044
Transcription factor Tubby signaling pathways	MetaCore	22	1.28E-03	0.048

**Table 2:** List of pathways designated as repressors of SREBP signaling by Wilcoxon test.

Pathway name	Pathway source	Probestes	Wilcoxon p-value	Wilcoxon FDR q-value
Extracellular matrix	PANTHER	136	2.97E-06	0.006
Cell adhesion_Cell-matrix interactions	MetaCore	89	8.85E-06	0.009
Extracellular matrix glycoprotein	PANTHER	41	1.84E-05	0.012
Skeletal development	PANTHER	54	6.14E-05	0.017
Serine protease	PANTHER	61	8.10E-05	0.017
Cell structure	PANTHER	238	8.60E-05	0.017
Cytoskeletal regulation by Rho GTPase	PANTHER	66	9.04E-05	0.017
Actin binding cytoskeletal protein	PANTHER	191	2.38E-04	0.023
Proteolysis_Connective tissue degradation	MetaCore	52	2.40E-04	0.023
Protocadherin alpha	PANTHER	9	2.48E-04	0.023
Myosin	PANTHER	31	3.52E-04	0.027
NF-kappaB cascade	PANTHER	17	3.52E-04	0.027
Serine protease related	PANTHER	25	6.23E-04	0.035
Microtubule family cytoskeletal protein	PANTHER	97	7.15E-04	0.036
Cytoskeleton_Cytoplasmic microtubules	MetaCore	71	7.42E-04	0.037
Urea cycle and metabolism of amino groups	KEGG	10	1.02E-03	0.040
Synthetase	PANTHER	41	1.03E-03	0.040
Proteolysis_ECM remodeling	MetaCore	38	1.08E-03	0.042

Application of a GSEA variant [51], the Levene test for homogeneity of variance as modified by Brown and Forsythe (LBF), [52] identified several pathways that included both positive and negative regulators of cholesterol homeostasis. The significant pathways identified once again included known regulators of cellular cholesterol homeostasis such as lipid metabolism, regulation of metabolism and Ras pathways as well as novel pathways such as Gap junction (Figure 3D), B-cell receptor and the Slit-Robo signaling pathways (Table 3). Notably, our screening results suggest a reciprocal relationship between gap junction formation and cholesterol homeostasis (see discussion and Table S3).

**Table 3:** List of SREBP pathway modulators by LBF test.

Pathway name	Pathway source	Probestes	LBF p-value	LBF FDR q-value
Gap junction	KEGG	52	3.00E-07	1.12E-04
Ras	PANTHER	8	9.81E-07	2.75E-04
Long-term depression	KEGG	32	1.38E-06	3.09E-04
Lipid metabolism	PANTHER	78	2.02E-06	4.11E-04
Zinc finger protein	PANTHER	19	3.77E-06	6.49E-04
Neoplasms, fibrous tissue	MetaCore	26	6.79E-06	1.01E-03
Neoplasms, connective and soft tissue	MetaCore	144	3.39E-05	4.22E-03
Cell adhesion_Platelet-endothelium-leucocyte interactions	MetaCore	76	5.22E-05	6.15E-03
Autoimmune diseases	MetaCore	189	5.58E-05	6.25E-03
Mucinoses	MetaCore	12	7.72E-05	8.23E-03
Plasmacytoma	MetaCore	60	8.87E-05	9.03E-03
Blood protein disorders	MetaCore	63	9.47E-05	9.22E-03
B-cell- and antibody-mediated immunity	PANTHER	33	1.21E-04	1.13E-02
Multiple myeloma/paraproteinemias	MetaCore	58	1.57E-04	1.15E-02
Hemorrhagic disorders	MetaCore	104	1.44E-04	1.15E-02
Anterior/posterior patterning	PANTHER	22	1.59E-04	1.15E-02
Mesoderm development	PANTHER	221	1.66E-04	1.16E-02
Neuroendocrine tumors	MetaCore	207	2.00E-04	1.32E-02
B cell receptor signaling pathway	KEGG	34	2.18E-04	1.36E-02
Macrophage-mediated immunity	PANTHER	46	2.65E-04	1.60E-02
Regulation of metabolism	MetaCore	135	3.64E-04	2.09E-02
Cell adhesion_Attractive and repulsive receptors	MetaCore	125	4.89E-04	2.70E-02
Slit-Robo signaling	MetaCore	29	5.06E-04	2.70E-02
Interleukin signaling pathway	PANTHER	66	4.96E-04	2.70E-02
Arachidonic acid production	MetaCore	7	7.20E-04	3.75E-02
Segment specification	PANTHER	31	8.43E-04	4.20E-02
Sarcoma	MetaCore	116	8.91E-04	4.29E-02
Vascular hemostatic disorders	MetaCore	73	9.01E-04	4.29E-02
Carcinoma, neuroendocrine	MetaCore	27	1.11E-03	4.87E-02

### Novel modifiers of SRE-luciferase act by stimulating or repressing SREBP activity

**Figure 4:**
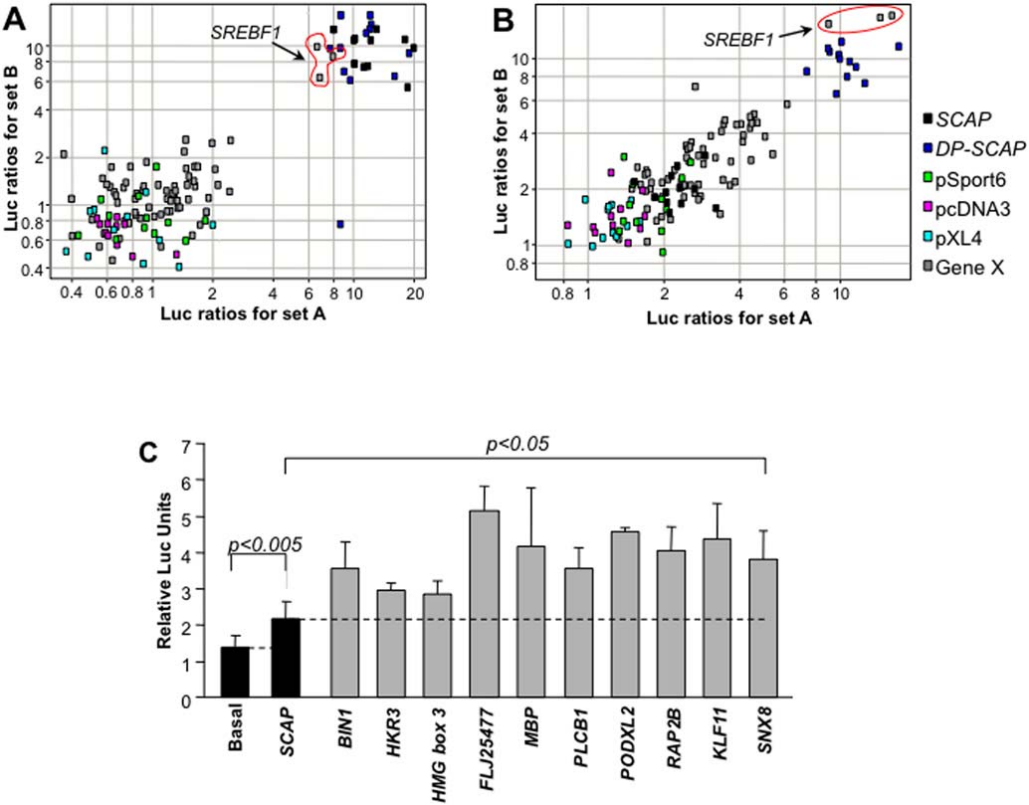
Novel activators of the SREBP pathway. (A) Effects of activator genes in presence of 25-OH cholesterol. The cDNA's were assayed in the presence of 1 µg/ml 25-hydroxy cholesterol. Scatter plot shows the luciferase ratios obtained for a clone in the first experiment against that obtained in the second experiment. Data points lying at the bottom left corner of the scatter plot represent genes that do not activate SRE-luciferase in the presence of high cholesterol. *SCAP* (black points), *DP-SCAP* (dark blue points) and *SREBF1* overcome cholesterol repression (top right corner). Empty vectors color coded as: green, pSport6; sky blue, pXL4; pink, pcDNA3. (B) Effects of *INSIG1* overexpression (15 ng/well) on novel activator genes. *SCAP* (central black points), DP-*SCAP* (dark blue points) and *SREBF1* (top right corner) escape repression by *INSIG1*. In addition, a number of candidate genes (grey points) activated SRE-luciferase to a greater extent than *SCAP*. Colored points represent empty vectors: green, pSport6; sky blue, pXL4; pink, pcDNA3. (C) Graphical representation of the ten novel activator genes that activate SREBP signaling under high INSIG1 levels. These genes activate the SREBP signaling assay to a significantly greater extent than SCAP (Student's t-Test, p<0.005 or p<0.05).

To further understand the influence of the candidate genes on regulating SREBP signaling, we tested the activators in the presence of excess sterol (1 µg/ml 25-OH cholesterol). As shown in Figure 4A, under high 25OH-cholesterol conditions, a majority of the genes identified as activators in our screen were clustered in the bottom left corner of the scatter plot indicating that the activity of these genes was attenuated in the presence of excess cholesterol. In contrast, we found only one gene (shown in triplicate) that was able to overcome high cholesterol levels (Figure 4A). This clone corresponded to sterol regulatory element binding protein 1c (*SREBP1c*).

In addition to excess cholesterol, we also tested the activators in the presence of high *INSIG1* levels to probe whether any of the genes in the activator set could overcome INSIG mediated pathway inhibition. As expected, co-overexpression of *SCAP* was found to overcome INSIG1-mediated SREBP stabilization. Of the 27 novel genes (Table S1) that promoted SREBP signaling, ten new genes were identified that were able to overcome the inhibitory effects of INSIG1 in a manner similar to that of SCAP, under conditions of excess INSIG1 (Figure 4B and 4C). These genes include bridging integrator-1 (*BIN1*), GLI-Kruppel family member, HKR3 (*HKR3*), high-mobility group box 3 (*HMG3*), the hypothetical protein FLJ25477, myelin basic protein (*MBP*), phospholipase C, beta 1 (*PLCB1*), podocalyxin-like 2 (*PODXL2*), RAP2B member of RAS oncogene family (*RAP2B*), kruppel-like factor 11 (*KLF11*) and sorting nexin 8 (*SNX8*).

**Figure 5:**
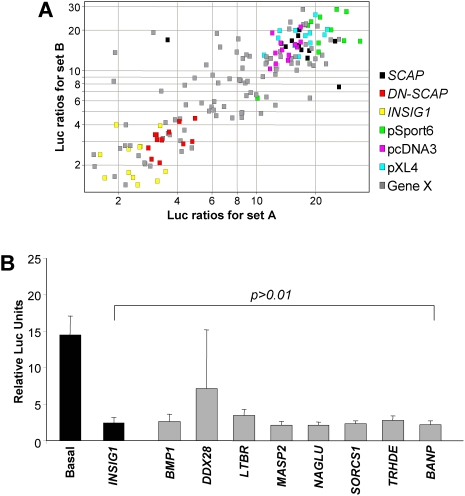
Novel repressors of the SREBP pathway. (A) Effects of *SCAP* (15 ng/well) over-expression on novel repressor genes. Scatter plot depicting the luciferase ratios obtained for a clone in the first experiment against ratios obtained in the second experiment. Internal control genes are color coded as: yellow, INSIG1; red, DN-SCAP; green, pSport6; sky blue, pXL4; pink, pcDNA3. (B) Graphical representation of eight repressors that can inhibit SCAP-mediated SREBP activation as efficiently as INSIG1 (Student's t-Test, p>0.01).

Activation of SREBP cleavage by over-expression of *SCAP* can be repressed by co-overexpression of *INSIG1* [40]. To examine if any of the 40 novel repressors could exert a similar effect as *INSIG1*, candidate repressors were tested for their ability to down-regulate elevated luciferase ratios resulting from *SCAP* overexpression. *INSIG1* and *DN-SCAP* could down-regulate SCAP induced SREBP signaling (Figure 5A, yellow and red points respectively) and served as controls. Interestingly, a number of candidate genes (grey points) localizing with *INSIG1* (yellow points) in the bottom left corner of the scatter plot were identified (Figure 5A), indicating these genes effectively repressed SREBP signaling despite *SCAP* over-expression. Eight genes repressed SCAP mediated activation of SREBP signaling as efficiently as INSIG1 (Figure 5B). These included bone morphogenetic protein 1 (*BMP1*), DEAD box polypeptide 28 (*DDX28*), lymphotoxin beta receptor (*LTBR*), mannan-binding lectin serine peptidase 2 (*MASP2*), N-acetyl-glucosaminidase, alpha (*NAGLU*), sortilin-related VPS10 domain containing receptor 1 (*SORCS1*), thyrotropin-releasing hormone degrading enzyme (*TRHDE*) and BTG3 associated nuclear protein (*BANP*).

To rule out the possibility that the effects of the novel genes identified in this screen are due to variations in transfection-control renilla luciferase levels, we have analyzed these values separately. We observe about a 2-fold variation in renilla luciferase values across the samples (Tables S1, S2). We believe that this variation is to be expected for a transient transfection experiment and does not influence the outcome of the luciferase assays significantly. The only cases where we have noticed the renilla luciferase values to be low are for the hypothetical protein FLJ25477 and RAP2B (Table S1).

## Discussion

Starting with a gene-by-gene approach to screen for modifiers of SRE-luciferase activity, we have identified several known and novel modulators of SREBP transcriptional activity.

With the aim of identifying novel activators of SREBP activity, we tested the primary hit list in the presence of high cholesterol and INSIG1 co-overexpression. Only one gene (*SREBF1*) was able to overcome these repressive conditions (Figure 4A and 4B). The activation of the SRE-luciferase reporter by SREBF1 even in the presence of sterols is most likely due to the production of the cleaved N-terminal transcriptional activator [14] . However, in the presence of INSIG1 co-overexpression, we identified ten novel genes that could overcome the inhibitory effects of INSIG1 (Figure 4C). Our finding that KLF11 and HMG3 act as SREBP modulators is in keeping with previous studies implicating these two classes of transcription factors in SREBP modulation [53]–[55]. Intriguingly, MBP, an integral component of myelin also activates SREBP signaling. A recent study implicates SREBP-1c and SREBP2 in the regulation of lipid metabolism and modulation of gene expression in Schwann cells, the myelinating cell of the peripheral nervous system [56]. Sorting nexin 8 (SNX8), another novel activator of SREBP signaling is a member of a diverse family of proteins that are grouped together based on the presence of a phospholipid binding Phox-homology (PX domain) that impact various intracellular trafficking and sorting events. Whether SNX8 regulates SREBP transcriptional activity by regulating intracellular trafficking events remains to be evaluated.

In contrast, by co-overexpression of SCAP, we have identified eight novel repressors of SREBP signaling (Figure 5B). Notably, recent results suggest that the immune system, through LTBR signaling, directly influences the enzymatic regulation of lipid homeostasis [57], underscoring the potential value of the novel modifiers identified in our screen. In addition, NAGLU is a lysosomal enzyme involved in the degradation of heparin sulfate [58]. Loss of NAGLU results in the lysosomal storage disease, Sanfilippo syndrome type B [58], [59]. SorCS1 is a type 1 transmembrane protein implicated in intracellular protein trafficking and sorting and predominantly localized to neurons [60], [61] and it is tempting to speculate that SorCS1 might play a role in lipid storage disorders of the brain. These novel repressors may mediate their effect via direct / indirect regulation of SCAP, via repressing SREBP transport or by modulating INSIG1 levels. Further characterization and validation studies are needed to distinguish between these possibilities and determine the precise mechanism of action of the identified repressors and activators.

With an aim to elucidate pathways involved in the coordinate control of SREBP signaling and cholesterol homeostasis we analyzed primary results from the gene by gene screen using a Gene Set Enrichment Analysis approach. In addition to known regulators, this analysis unraveled novel roles for several pathways including the ephrin receptor (EphR) and EGF receptor (EGFR) signaling pathways, as putative activators or inhibitors of SREBP signaling. Both EGFR and EphR have been shown to associate with caveolin-1 positive microdomains [62], [63] and signal via cholesterol-rich lipid rafts, implying that these pathways might be positively regulated by cellular cholesterol levels. Also, receptor tyrosine kinases such as EGFR and EphRs activate MAPK signaling which has been shown to stimulate SREBP transcriptional activity [64]. Signaling via the transcription factor Tubby represents another novel positive regulator of cholesterol homeostasis identified in our screen (Table 1). Tubby has been shown to bind to plasma membrane phophoinositides and participate in heterotrimeric G-protein coupled receptor signaling (GPCR) and there is ample evidence in support of a crucial role for cholesterol in modulating GPCR function (reviewed in [65]). It is worth noting that recent studies have also unraveled a novel role for tubby in regulating a Rab-dependent endocytic trafficking pathway [66]. Enrichment for serine proteases as gathered from our GSEA results indicates that in addition to the critical S1P/S2P mediated cleavage of SREBP, and PCSK9 catalyzed LDLR trafficking [67], [68], additional important nodes in the SREBP signaling pathway might be regulated by proteases. Also, the notion that efficient and directional intracellular trafficking of vesicles is closely dependent on microtubule and cytoskeleton dynamics is supported by our analysis where we have identified associated pathways as modifiers.

Finally, using a series of biological validation assays we successfully matched number of GSEA significant pathways to the genes identified in our screen (Table 4). It is worth noting that the genes validated in this study, namely the activators *RAB20*, *RAB8A* and *BIN1* [69], [70] and the repressors, SNX8 [71], [72] and SorCS1 [60] have all been implicated in membrane and vesicle trafficking events. Interestingly, a recent gene expression profiling comparison of normal and Niemann Pick disease type C (NPC) patient fibroblasts revealed changes in several genes important for membrane traffic including *RAB20* and SorCS1 [73]. The LBF GSEA analysis additionally points to a potential link between gap junction formation and cholesterol homeostasis (Table S3). Our screening results indicate that growth factors [74], protein kinase A, and protein kinase C [75]–[77] signaling cascades that dampen the formation and/or function of gap junctions also induce SREBP activity. Conversely, microtubule related tubulins involved in hemichannel transport [78], and casein kinases which promote the formation of gap junctions [75], [77], [79] , inhibit SREBP activity in our screen. Taken together, these results suggest a reciprocal relationship between gap junction formation and cholesterol biosynthesis that may warrant further investigation. The novel genes and pathways identified in the screen and subsequent pathway analysis detailed in this study, when validated in disease relevant contexts could represent novel therapeutic entry points or pathway nodes that enhance our understanding of lipid biology.

**Table 4:** Overlap between GSEA and biological validation.

Pathway name from GSEA	Gene from biological analysis
Regulation of metabolism	*SREBF1*, *INSIG2*, *ACSL4*
Cell adhesion_Platelet-endothelium-leucocyte interactions	*SCARF1*
Serine protease	*LACTB*, *ABHD4*, *MASP2*
n-3,6 Polyunsaturated & unsaturated fatty acid biosynthesis	*ACSL4*
Arachidonic acid production	*ACSL4*
G-protein, small GTPase & RAS-related GTPase	*RAB20*, *RAB8A*, *RAP2B*
Neuroendocrine tumors	*BIN1*, *RAB8A*
Lipid & sphingolipid metabolism	*SPTLC1*, *PLCB1*
Autoimmune diseases	*MBP*, *SPTLC1*
Cell structure & Gap junction	*CSNK1E*, *DGCR14*, *MBP*

Novel genes regulating SREBP signaling that were identified in the biological re-screens were mapped onto the pathway analysis data. Shown here are the novel genes that showed up in both biological as well as pathway analyses.

## Materials and Methods

### Reporter constructs

Three copies of the sterol regulatory element (SRE, AAAATCACCCCACTGCAAACTCCTCCCCCTGC) from the low-density lipoprotein receptor gene promoter [18] were subcloned upstream of a pTransLucent (Panomics) and pGL3-Basic (Promega) luciferase vector to create a SRE-luciferase plasmid. As a control, a mutated version of this promoter was synthesized (Medigenomics) to contain four point mutations in each SRE element as previously reported [49] (AAAAGAACCCCTATGCAAACTCCTCCCCCTGC, mutations underlined). As an internal transfection normalization control, a humanized renilla luciferase gene driven by a weak ubiquitous SV-40 promoter (phRL-SV40, Promega) was used.

### Plasmids and human cDNA clone collection

The cDNA used as controls for the SREBP cleavage assay, namely full length hamster SREBP-cleavage activating protein (SCAP), human INSIG1, hamster dominant positive and dominant negative SCAP (DP-SCAP/DN-SCAP) have been previously described [18], [40], [50], [80]. For the genome-wide study, approximately 10,000 full-length cDNA clones were purchased from OriGene Technologies (Rockville, MD) and prepared for screening as previously described [81].

### Cell lines and growth conditions

CHO wild-type and HEK-293 cells were obtained from ATCC. The CHO wild-type and mutant cell lines were grown in F-12 (HAM) media (Invitrogen), supplemented with 5% new-born calf serum (NCS), 10 mM HEPES buffer and 1× Penicillin-Streptomycin (Invitrogen) antibiotic. HEK-293 cells were grown in DMEM (Invitrogen) supplemented with 10% fetal bovine serum (Invitrogen) and containing 1× Penicillin-Streptomycin (Invitrogen). All cell lines were grown in a humidified incubator at 37°C and with 5% CO_2_. 25-hydroxy (25-OH) cholesterol (Sigma) was dissolved added to media as indicated in figure legends.

### Genome-wide cDNA study and luciferase assays

A reverse transfection protocol was followed for testing the 10,000 genes for their effect in the SREBP signaling assay. Trypsinized HEK-293 cells were added to 384-well white opaque bottom plates (Nunc), containing the cDNA clone and transfection mix, at density of 2500 cells/well at 25 µl per well using a Multidrop 384 (Thermo Labsystems) and incubated at 37°C in 5% CO_2_. The transfection mix consisted of 17.5 ng reporter plasmid/well, 0.7 ng Renilla/well and Fugene 6 (Roche) at a ratio of 3 µl Fugene to 1 µg of DNA in 5 µl of Optimem (Invitrogen). The transfection mixture was added to the 384-well plates containing the cDNA clone using a FlexDrop (Perkin Elmer). The cDNA's were screened at a concentration of 120 ng/well. For cholesterol stimulation, 25-hydroxycholesterol (Sigma) in 0.01% ethanol was added to cells at a concentration of 1 µg/ml, and incubated for 24 hours. Firefly and Renilla luciferase activity was measured 40 hours post transfection using the Dual Glow assay system (Promega). Plates were allowed to cool for 10 minutes before 30 µl of each assay reagent was added. The plates were shaken for 10 minutes on a multi-plate shaker. Luminescence was determined using an EnVision plate reader (Perkin Elmer) with a 100 msec integration time. For luciferase assays carried out in 96-well plates, all reagents were proportionally increased 4-fold.

### Data analysis and 2D-normalization of genome-wide study

Results were analyzed using Spotfire Decisionsite software and Microsoft Excel. For the genome-wide study, luciferase ratios were normalized as follows. The firefly-luciferase readout values were first normalized using the corresponding renilla-luciferase readout values. The firefly-renilla ratio was further normalized as follows. The one-dimensional (1D) values were obtained by scaling the ratios with the plate median in order to remove plate-to-plate variation. The two-dimensional (2D) values were obtained by further removing the well-to-well variations through an iterative procedure. Finally, the normalized values were standardized to obtain the NZ score, which is a more robust equivalent of the more commonly used Z score. If the distribution is perfectly normal, NZ score will be the same as the Z score.

### GSEA methodology

Screening results were analyzed with a modified version of the Gene Set Enrichment Analysis (GSEA) technique previously described elsewhere [47], [51], [82]. As input, 2D normalized z-scores (NZ2D) were first computed to estimate the effect of each cDNA on the SREBP assay readout. NZ2D scores were averaged per cDNA across replicates. These averaged NZ2D values were used to rank the cDNAs for input to the GSEA method. Two variants of the GSEA method were applied to these ranked scores. The first method represented the standard GSEA approach [47], [82] and used the Wilcoxon ranked sum test [83] to identify pathways whose members tended to activated or inhibited the assay. The second GSEA variant applied a robust test for homogeneity of variance [51], the Levene test as modified by Brown and Forsythe (LBF) [52]. Application of the LBF test was used to identify pathways that contain similar numbers of activators and repressors of the assay. Such cases may elude detection by the Wilcoxon test, as the contributions of activators and inhibitors tend to cancel each other out. The presence of activators and inhibitors within a pathway will yield a larger variance of NZ2D scores than is generally present in the assay and is thus detectable by the LBF test. Finally, a false discovery rate (FDR) [84] correction was applied to the computed p-values to account for multiple hypothesis testing. This process transforms the original p-values into FDR q-values that were used for significance testing. The GSEA results were then filtered to identify interesting pathways by 1) removing pathways with <5 clones; 2) removing pathways with >250 clones; 3) removing pathways with FDR q-values>0.05 for the Wilcoxon and LBF tests. This resulted in 103 moderately-sized pathways that had hits at q-values<0.05 in at least one test.

This application of GSEA is a natural extension of a methodology that has enjoyed great success when applied to microarray data [47], [51], [82]. Nevertheless, there are fundamental differences between these types of experiments that impact the interpretation of results. Whereas a simple microarray experiment consists of a single perturbation and readouts for tens of thousands of genes, this screen includes thousands of cDNA overexpression perturbations and a single readout. When applied to microarray data, GSEA identifies pathways that are modulated in response to a specific perturbation. In this application, GSEA should identify pathways that modulate SREBP activity. The recovery of several pathways known to modulate cholesterol homeostasis validates the application of pathway-centric methodologies for analyzing cDNA overexpression screens.

## Supporting Information

Figure S1 Scatter plot of the novel activators (red) and repressors (blue) of SREBP signaling after removal of the false positives and clones with high renilla luciferase levels.
(1.31 MB TIF)

Table S1 Complete list of validated activators of SREBP signaling.
(0.04 MB XLS)

Table S2 Complete list of validated repressors of SREBP signaling.
(0.04 MB XLS)

Table S3 Genes that affect the formation and function of Gap Junctions also modulate SREBP activity: Several branches of the KEGG Gap Junction (HSA04540) pathway were notable for their coordinate regulation of the SREBP assay. Notably, positive regulators of gap junctions tended to inhibit SREBP activity (Casein Kinases, Microtubules), whereas negative regulators tended to activate SREBP (Growth Factors, Protein Kinase C, Protein Kinase G). The most potent inhibitor and activator among these were validated in secondary assays (PLCB1, CSNK1E). Some genes showed isoform selectivity for SREBP activity, e.g. the epsilon isoform of casein kinase 1 was a more potent activator than the delta and gamma isoforms. Nevertheless, the consistency of results across several independent branches of gap junction formation signaling pathways suggests an inverse causal relationship between the function of gap junctions and cholesterol homeostasis.
(0.03 MB XLS)
