# Supplementary figures and images for: Identification of Novel Genes and Pathways Regulating SREBP Transcriptional Activity
Source: PLoS One. 2009 Apr 21;4(4):e5197. doi: 10.1371/journal.pone.0005197 (PMC2668173; doi:10.1371/journal.pone.0005197)

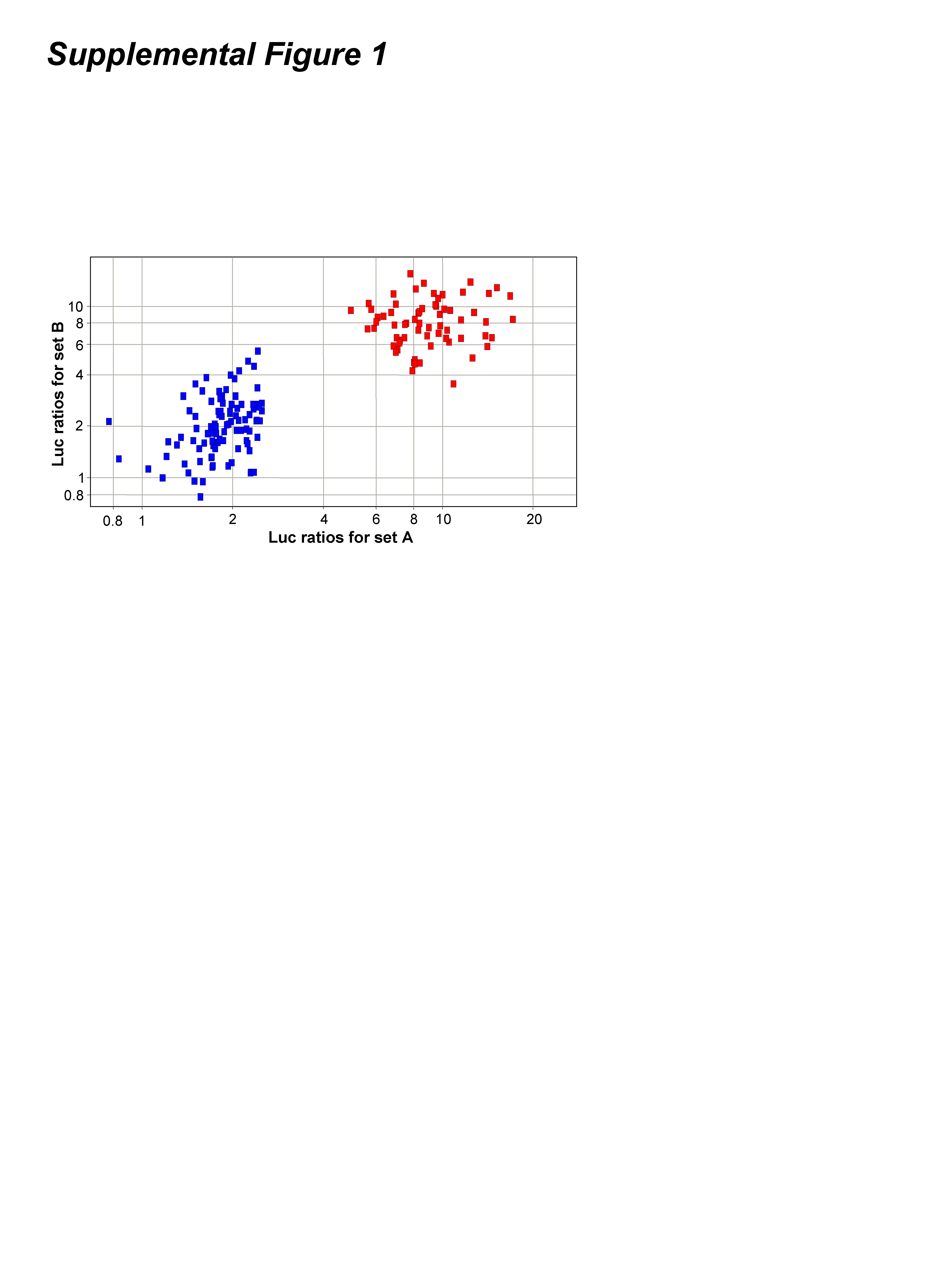

Supplement: Figure S1 — Scatter plot of the novel activators (red) and repressors (blue) of SREBP signaling after removal of the false positives and clones with high renilla luciferase levels. (1.31 MB TIF) [file pone.0005197.s001.tif]
